# Correction and Republication: Abortion Surveillance — United States, 2014

**DOI:** 10.15585/mmwr.mm6746a5

**Published:** 2018-11-23

On November 24, 2017, *MMWR* published “Abortion Surveillance — United States, 2014” (*1*). On August 6, 2018, the authors informed *MMWR* about inadvertent errors in the data that resulted in publication of some erroneous numbers for gestational ages and abortion ratios throughout the report. The authors have corrected these errors and confirm that the interpretation or the conclusions of the original report have not changed. Additional text has been added to clarify how CDC adjusts gestational age data. In accordance with December 2017 guidance from the International Committee of Medical Journal Editors (*2*), *MMWR* is republishing the corrected report. The republished report has supplementary materials that include the original report with these corrections and additional text clearly marked (*3*).
